# Orexin/Hypocretin and Histamine Cross-Talk on Hypothalamic Neuron Counts in Mice

**DOI:** 10.3389/fnins.2021.660518

**Published:** 2021-05-20

**Authors:** Chiara Berteotti, Viviana Lo Martire, Sara Alvente, Stefano Bastianini, Cristiano Bombardi, Gabriele Matteoli, Hiroshi Ohtsu, Jian-Sheng Lin, Alessandro Silvani, Giovanna Zoccoli

**Affiliations:** ^1^PRISM Lab, Department of Biomedical and Neuromotor Sciences, Center for Applied Biomedical Research, S. Orsola University Hospital, Alma Mater Studiorum–University of Bologna, Bologna, Italy; ^2^Department of Veterinary Medical Sciences, University of Bologna, Bologna, Italy; ^3^Tekiju Clinic, Nagataku, Kobe, Japan; ^4^Physiologie Intégrée du Système d’éveil, Centre de Recherche en Neurosciences de Lyon, INSERM U1028-CNRS UMR, Bron, France

**Keywords:** orexins/hypocretins, histamine, neurons, narcolepsy, mouse

## Abstract

The loss of hypothalamic neurons that produce wake-promoting orexin (hypocretin) neuropeptides is responsible for narcolepsy type 1 (NT1). While the number of histamine neurons is increased in patients with NT1, results on orexin-deficient mouse models of NT1 are inconsistent. On the other hand, the effect of histamine deficiency on orexin neuron number has never been tested on mammals, even though histamine has been reported to be essential for the development of a functional orexin system in zebrafish. The aim of this study was to test whether histamine neurons are increased in number in orexin-deficient mice and whether orexin neurons are decreased in number in histamine-deficient mice. The hypothalamic neurons expressing L-histidine decarboxylase (HDC), the histamine synthesis enzyme, and those expressing orexin A were counted in four orexin knock-out mice, four histamine-deficient HDC knock-out mice, and four wild-type C57BL/6J mice. The number of HDC-positive neurons was significantly higher in orexin knock-out than in wild-type mice (2,502 ± 77 vs. 1,800 ± 213, respectively, one-tailed *t*-test, *P* = 0.011). Conversely, the number of orexin neurons was not significantly lower in HDC knock-out than in wild-type mice (2,306 ± 56 vs. 2,320 ± 120, respectively, one-tailed *t*-test, *P* = 0.459). These data support the view that orexin peptide deficiency is sufficient to increase histamine neuron number, supporting the involvement of the histamine waking system in the pathophysiology of NT1. Conversely, these data do not support a significant role of histamine in orexin neuron development in mammals.

## Introduction

The interactions between the orexin (hypocretin) (de Lecea et al., 1998; Sakurai et al., 1998) and histamine neuron systems are an open topic of investigation of substantial scientific and potential clinical relevance (Sundvik and Panula, 2015). The loss of hypothalamic neurons that release wake-promoting orexin/hypocretin neuropeptides is the main pathophysiological event leading to narcolepsy type 1 (NT1) (Peyron et al., 2000; Thannickal et al., 2000). Histamine neurons of the tuberomammillary nucleus (TMN), another wake-promoting system (Sundvik and Panula, 2015), are excited by orexin neurons (Eriksson et al., 2001). Two independent studies (John et al., 2013; Valko et al., 2013) reported an increased number of histamine neurons at autopsy in the brains of patients with NT1. These results keep open the question whether orexin peptide deficiency is sufficient to increase histamine neuron number, given that patients also lose orexin co-transmitters (Bonnavion et al., 2016) and may suffer from consequences of an autoimmune insult to the hypothalamus (Kornum, 2020). Contrasting results obtained by the same studies (John et al., 2013; Valko et al., 2013) on mice did not help to clarify the issue. In particular, one study (Valko et al., 2013) found a significant 53% increase in histamine neurons in adult orexin knock-out mice (KO-ORX) compared to wild-type (WT) control mice, whereas the other study (John et al., 2013) did not find significantly altered numbers of histamine neurons.

The question of whether histamine deficiency impacts on orexin neuron number is also still open. Some data obtained in larval zebrafish indicated that orexin neuron number is decreased with histamine deficiency and increased with histamine excess (Sundvik et al., 2011), but this finding was not replicated in later work (Chen et al., 2017). Moreover, the effects of histamine deficiency on orexin neuron number have not been tested on mammals yet. Interestingly, however, total brain orexin levels measured by enzyme immunoassay were found lower in histamine receptor 1 knockout mice than in WT control mice (Lin et al., 2002).

The aim of this study was to test whether histamine neurons are increased in number in orexin-deficient KO-ORX mice and whether orexin neurons are decreased in number in histamine-deficient mice.

## Materials and Methods

The study protocols complied with the EU Directive 2010/63/EU and with Italian law (DL 26, March 4, 2014) and were approved by the Italian Ministry of Health (protocols n. 245/2015-PR and 141/2018-PR).

The hypothalamus of the following 12 adult male mice was examined: four homozygous KO-ORX mice congenitally lacking orexin peptides (Chemelli et al., 1999), four homozygous L-histidine decarboxylase (HDC) knock-out (KO-HIST) mice congenitally lacking HDC, the enzyme for histamine synthesis (Ohtsu et al., 2001), and four WT mice, as controls. Mice of the different groups were not littermates. The mating schemes adopted for KO-HIST and KO-ORX mice (homozygous × homozygous or homozygous × heterozygous) were designed to maximize the yield of homozygous mice but did not yield littermate WT mice. The KO-HIST, KO-ORX, and WT mice were fully congenic to C57Bl/6J mice (*N* > 10 generations of backcrossing). Mice were raised at 25°C with a 12:12 h light-dark cycle and free access to food and water. Mouse genotyping was performed as previously described (Bastianini et al., 2011, 2015).

Mice were euthanized in adult age (KO-HIST: 43 ± 1 weeks; KO-ORX: 39 ± 1 weeks; WT: 51 ± 5 weeks) 2–4 h after the beginning of the light period, i.e., at Zeitgeber time (ZT) 2–4. Under deep anesthesia, mice were perfused with saline followed by 4% paraformaldehyde. Brains were cryoprotected in PBS with 20% sucrose and coronally sectioned at 30 μm using a cryostat-microtome at –22.0°C (Frigocut 2800) into a 1:3 series. Free-floating sections were washed in 0.3% Triton X-100 in PBS for 30 min. After blocking for 90 min with 3% bovine serum albumin (Sigma Aldrich, Milan) in 0.3% Triton X-100 in PBS, sections were incubated overnight at 4°C with either anti-HDC or anti-orexin A primary antibodies. The anti-HDC antibody was a rabbit anti-HDC antiserum raised against amino acids 1–481 of the rat HDC peptide (catalog # 03–16045, American Research Products, Waltham, MA, United States) diluted 1:2,500 in 0.3% Triton X-100 in PBS and BSA 1%. The anti-orexin antibody was a rabbit anti–orexin A antiserum (catalog H-003-30, Phoenix Pharmaceuticals, Burlingame, CA, United States) diluted 1:5,000 in 0.3% Triton X-100 in PBS and BSA 1%. Sections were then washed in 0.3% Triton X-100 in PBS for 30 min and incubated for 2 h with a Cy3-conjugated AffiniPure Donkey Anti-rabbit IgG (Jackson ImmunoResearch, West Grove, PA, United States) secondary fluorescent antibody, diluted 1:200 in 0.3% Triton X-100 in PBS and BSA 1%. Slides were finally cover-slipped with glycerol buffer and stored at –20°C in the dark. Sections were counterstained with Hoechst 33342 (Sigma-Aldrich) to label cell nuclei. Immunofluorescence images were captured with a Nikon Eclipse TE 2000-S inverted microscope (Nikon Corp., Kawasaki, Japan; final magnification: 100× or 200×) equipped with a Nikon digital camera (model DS-Qi2). For the sole purpose of figure publication, the contrast and brightness of the figures were adjusted to resemble the appearance of the labeling seen through the microscope using Adobe Photoshop CS3 Extended 10.0 software (Adobe Systems, San Jose, CA, United States).

L-histidine decarboxylase and orexin A neurons were counted using Image J software 1.50i^1^. For HDC positive neuron counts, care was taken to refer to the reported boundaries of the TMN in mice (Franklin and Paxinos, 2007). For the sake of comparison with previous published work (John et al., 2013; Valko et al., 2013), boundaries of 20 positively labeled neurons per section were manually delineated to measure cell size at a final magnification of 200×. Only neurons identified by their intense immunoreactivity and the presence of processes were analyzed. Cells that were out of focus were not evaluated.

Neurons positive to HDC or to orexin A were quantified on every third section throughout the hypothalamus. The total number of HDC- and orexin A− positive neurons per brain was estimated by multiplying the number of cells counted in the series of sampled sections by 3, which corresponded to the section sampling frequency, and applying the Abercrombie correction (Abercrombie, 1946) with the following formula: actual number = (raw number × section thickness)/(section thickness + cell diameter), where section thickness was 30 μm and average cell diameter was calculated from the measured cell size. Staining for HDC and orexin A was performed also in a subset of KO-HIST and KO-ORX mouse brain sections, respectively, as a test for antibody specificity. The same investigator (CBe), blinded to the experimental group, performed all counts. To evaluate the location of HDC-positive neurons in the TMN, each section that contained HDC-positive cells in the TMN was assigned to one of three groups with reference to the mouse brain atlas (Franklin and Paxinos, 2007): >2.6, 2.6–2.2, and <2.2 mm caudal to bregma. To evaluate cell size distribution between different genotypes, the relative frequency of HDC- or orexin-A positive cell size was computed for each animal, then averaged over animals of each genotype.

Statistical analysis was performed using SPSS software (SPSS Inc., Chicago, IL, United States) or GraphPad Prism (version 7). The sample size of KO-ORX and WT mice was determined with an *a priori* statistical power analysis to yield a statistical power >80% with a type-I error rate α of 0.05 and one-tailed *t*-test, based on data reported from Valko et al. (2013) and the directional hypothesis of more histamine neurons in KO-ORX than in WT. The statistical power analysis was performed with the *G*^∗^power free application (Faul et al., 2007). In the absence of previous data on mice, the same effect size was assumed and, thus, the same sample size was adopted for an exploratory test of the directional hypothesis of fewer orexin neurons in KO-HIST mice than in WT (Sundvik et al., 2011). To summarize, we chose *a priori* to test specific directional hypotheses (more histamine neurons in KO-ORX than in WT mice; fewer orexin neurons in KO-HIST than in WT mice) based on the empirical evidence on human subjects, mice, and zebrafish available at the time of the study (Sundvik et al., 2011; John et al., 2013; Valko et al., 2013). Our statistical design was adequate to support or not support these hypotheses but was inadequate to demonstrate any unanticipated effect in the untested directions. By focusing on testing hypotheses based on prior empirical evidence, this choice allowed us to limit the sample size (i.e., the number of animals used for research) needed to achieve the desired statistical power.

Results are shown as mean ± SEM with significance at *P* < 0.05 with one-tailed independent-sample *t*-tests for cell number. Absent prior directional hypotheses, two-tailed independent-sample *t*-tests were performed on mouse age and cell size for control purposes. The correlation between mouse age and either orexin- or HDC-positive cells for each mouse group was assessed by computing Pearson’s correlation coefficients.

The anteroposterior location of HDC-positive cells in TMN was assessed with two-way ANOVA with distance from bregma (>2.6, 2.6–2.2, and <2.2 mm caudal to bregma) and mouse genotype (two levels: WT vs. KO-ORX) as factors. In case of significance of the two-way interaction, simple effects of genotype were assessed with one-tailed independent-sample *t*-tests with Bonferroni correction for multiple comparisons.

The HDC- or orexin-A positive cell size distributions were assessed with two-way ANOVA (Lemus et al., 2015; Hyeun et al., 2019) with HDC- or orexin-A positive cell size (five levels: 50 μm^2^ bins from 100 to 350 μm^2^ for HDC-positive cell size and from 150 to 400 μm^2^ for orexin-A-positive cell size; bin range set to avoid empty bins) and mouse genotype (two levels: WT vs. KO-ORX or KO-HIST) as factors and with the mouse as the experimental unit. The normality ANOVA assumption was verified with one-sample Kolmogorov Smirnov tests as appropriate.

## Results

As expected, the HDC antiserum produced no staining in the TMN of KO-HIST mice, but it labeled presumably monoamine neurons in *substantia nigra*, ventral tegmental area, and dorsal raphe nuclei, perhaps by cross-reacting with aromatic L-amino acid decarboxylase (Mizuguchi et al., 1990). The orexin A antiserum produced no staining in the brains of KO-ORX mice.

**FIGURE 1:**
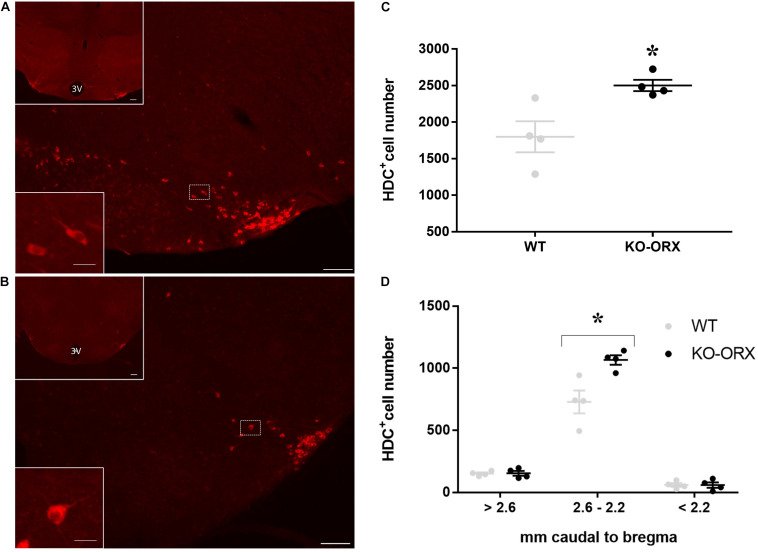
L-histidine decarboxylase-positive cells in orexin knockout and wild-type mice. **(A,B)** Show representative sections of L-histidine decarboxylase (HDC) immunostaining (cells with red cytoplasm) in orexin knockout (KO-ORX), and control wild-type (WT) mice, respectively. In both panels, the bottom left insets show a higher magnification of the delimited area (white square) in the main panel. 3V: third ventricle. Scale bars = 100 μm for main panels and upper insets and 12.5 μm for the lower insets. For the sole purpose of figure publication, the contrast, and brightness were adjusted to resemble the appearance of the labeling seen through the microscope using Adobe Photoshop CS3 Extended 10.0 software (Adobe Systems, San Jose, CA, United States). **(C)** Shows the total number of HDC- positive neurons in four WT control and four KO-ORX mice. **(D)** Shows the number of HDC-positive neurons in four KO-ORX and four WT mice as a function of distance from bregma along the rostrocaudal axis, with reference to the mouse brain atlas (Franklin and Paxinos, 2007). Gray and black dots represent individual animals. The horizontal line and whiskers indicate means ± SEM. *P < 0.05 vs. WT, one-sample independent *t*-test.

Representative sections at different magnifications showing HDC-positive neurons in KO-ORX and WT mice are reported in Figures 1A,B. HDC and orexin A in mouse brains was quite dense and clearly contrasted with the background, with well-defined somata, proximal dendrites, and axons. The distributions of the number of HDC-positive neurons did not differ significantly from the normal distribution (*P* ≥ 0.867, one-sample Kolmogorov-Smirnov test). The estimated total number of HDC-positive TMN neurons per brain was 39% higher in KO-ORX than in WT mice (*P* = 0.011, one-tailed *t*-test) (Figure 1C), and the increase in KO-ORX mice remained significant when assessed on raw neuron counts without the Abercrombie correction (1279 ± 40 vs. 940 ± 111, *P* = 0.014, one-tailed *t*-test). Age did not differ significantly between KO-ORX and WT mice (*P* = 0.101, two-tailed *t*-test) and did not correlate significantly with the number of HDC-positive TMN neurons in either group (*P* ≥ 0.390, Pearson’s correlation). The anteroposterior location of HDC-positive neurons in the TMN evidenced a significant interaction between distance from bregma along the rostro-caudal axis and mouse group (*P* = 0.002, two-way ANOVA) (Figure 1D). KO-ORX mice had more HDC-positive TMN neurons than WT mice in the interval between 2.6 and 2.2 mm caudal to bregma (*P* < 0.001, one-tailed *t*-test). The sections located >2.6 or <2.2 mm caudal to bregma did not evidence a significant increase in HDC-positive neuron count in KO-ORX with respect to WT mice (*P* ≥ 0.999, one-tailed *t*-tests).

**FIGURE 2:**
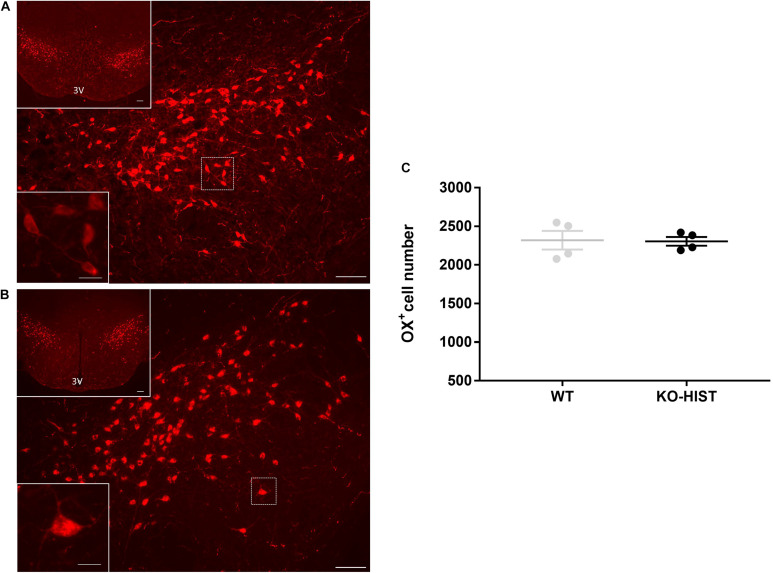
Orexin-A positive cells in L-histidine decarboxylase knockout and wild-type mice. **(A,B)** Show representative sections of orexin immunostaining (cells with red cytoplasm) in L-histidine decarboxylase knockout mice (KO-HIST) and WT mice, respectively. In both panels, the bottom left insets show a higher magnification of the delimited area (white square) in the main panel. 3V: third ventricle. Scale bars = 100 μm for main panels and upper insets and 12.5 μm for the lower insets. For the sole purpose of figure publication, the contrast and brightness were adjusted to resemble the appearance of the labeling seen through the microscope using Adobe Photoshop CS3 Extended 10.0 software (Adobe Systems, San Jose, CA, United States). **(C)** Shows the total number of orexin-positive neurons in four WT control and four KO-HIST mice. Gray and black dots represent individual animals. The horizontal line and whiskers indicate means ± SEM.

Representative sections at different magnifications showing orexin-positive neurons in KO-HIST and WT mice are shown in Figures 2A,B. The distributions of the number of orexin-positive neurons did not differ significantly from the normal distribution (*P* ≥ 0.915, one-sample Kolmogorov-Smirnov test). The estimated total number of orexin neurons per brain was not significantly lower in KO-HIST than in WT mice (*P* = 0.460, one-tailed *t*-test) (Figure 2C). Age did not differ significantly between KO-HIST and WT mice (*P* = 0.812, two-tailed *t*-test).

**FIGURE 3:**
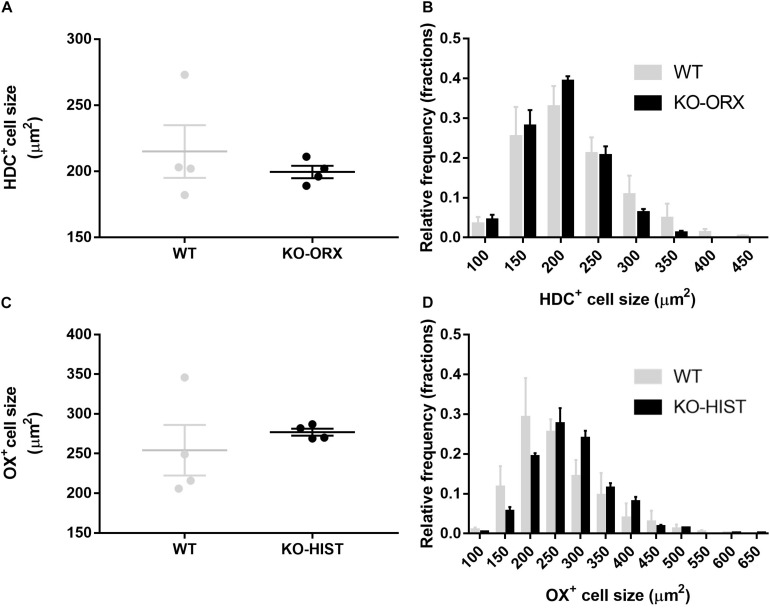
L-histidine decarboxylase-positive and orexin-A positive cell size. **(A)** Shows HDC-positive average cell size in four WT mice and four KO-ORX mice. **(C)** Shows average orexin-A positive cell size in four WT mice and four KO-HIST mice. Gray and black dots represent individual animals. **(B)** Shows the HDC-positive cell size distribution (50 μm^2^ bins, X axis) in four WT (gray) mice and four KO-ORX (black) mice. **(D)** Shows the orexin-A positive cell size distribution (50 μm^2^ bins, X axis) in four WT (gray) mice and four KO-HIST (black) mice. **(A,C)** The horizontal line and whiskers indicate means ± SEM. **(B,D)** Data are reported as mean + SEM.

The cell size of HDC-positive TMN neurons did not differ significantly between KO-ORX and WT mice (*P* = 0.478, two-tailed *t*-test) (Figure 3A). The resulting estimated mean cell diameters for the Abercrombie correction were 16 and 17 μm, respectively. The cell size of orexin-A neurons did not significantly differ between KO-HIST and WT mice, either (*P* = 0.507, two-tailed *t*-test) (Figure 3C). The resulting estimated mean cell diameters for the Abercrombie correction were 19 and 18 μm, respectively. Accordingly, neither the distribution of HDC-positive cell size (Figure 3B) nor that of orexin-A positive cell size (Figure 3D) evidenced a significant interaction between cell size and mouse group (*P* = 0.638 and *P* = 0.463, respectively, two-way ANOVA).

## Discussion

The number of HDC-positive cells in the TMN was 39% higher in KO-ORX compared to WT mice, with no difference in the mean cell size. The number of orexin neurons was not significantly lower in KO-HIST than in WT mice, and their mean cell size did not differ significantly, either. The increase in the number of HDC-positive cells in the TMN in KO-ORX mice compared to WT controls was in accordance with results obtained in patients with NT1 (John et al., 2013; Valko et al., 2013), and this increase seemed to be localized in the central part of the TMN, confirming a previous contested finding on KO-ORX mice (Valko et al., 2013). However, we found a smaller increase of HDC-positive cells in KO-ORX mice compared to WT with respect to previously published work (Valko et al., 2013). This difference could be due to biological variability, to different immunohistochemical procedures, i.e., use of avidin/biotin complex and nickel-enriched 3,30-diaminobenzidine in previous work (Valko et al., 2013) versus immunofluorescence in our study, or to mouse sacrifice timing, which was not reported in previous work (Valko et al., 2013) and occurred at ZT2-4 in our study. This last difference could be crucial: the number of HDC-positive cells significantly increased (+34%) in mice sacrificed during the dark period (ZT17) compared to the light period (ZT5, McGregor et al., 2017). It would be interesting, in future experiment, to analyse the difference in the number of HDC-positive cells between KO-ORX and WT mice at different ZT.

Our finding on KO-ORX mice fully congenic (*N* > 10 generations of backcrossing) to the C57Bl/6J genetic background thus confirmed a previous published report on KO-ORX mice that were also highly congenic (*N* = 8–10 generations of backcrossing) to C57Bl/6J (Valko et al., 2013). Our findings may suggest that the negative result published by another study (John et al., 2013) on hybrid C57BL/6J-129/SvEv mice was either a type II statistical error or a result of differences in genetic background. Regardless, our data support the view that at least on the C57Bl/6J background, congenital orexin peptide deficiency is sufficient to increase histamine neuron number in adulthood. This result may represent a homeostatic compensation for the loss of orexin peptides, or for an impairment of histaminergic neurotransmission, recently described in narcoleptic children close to disease onset (Franco et al., 2019).

Our data do not inform on whether the additional HDC-positive neurons in KO-ORX mice resulted from enhanced differentiation of HDC-positive neurons during early development, from adult neurogenesis and differentiation, or from increased expression of HDC that brought a population of existing HDC-expressing neurons above the threshold for immunohistochemical detection. In favor of the last hypothesis, as previously mentioned, recent data indicate that HDC-positive neuron number increases 34% from the light to the dark period in mice, without changes in cell size (McGregor et al., 2017). A subpopulation of “non-visible” phenotypically defined cells also characterizes orexin neurons, as mice also showed 24% more orexin-positive neurons during the dark than during the light period, again without changes in cell size (McGregor et al., 2017).

We did not detect a significant decrease in the number of orexin neurons in KO-HIST mice compared to WT mice. This is at variance with previous results in developing zebrafish, in which a decrease in histamine signaling has been associated to a reduction in the number of orexin neurons (Sundvik et al., 2011). However, our negative findings are in line with the results of a later study, which reported that the number of orexin neurons was unaffected in zebrafish larvae lacking histamine (Chen et al., 2017). Our study did not address the possibility that orexin neuron development may be affected by histamine co-transmitters.

Our study has limitations. The sample size was small and limited to four mice per group. However, we estimated the sample size of KO-ORX and WT mice with an *a priori* power analysis based on the directional hypothesis of more histamine neurons in KO-ORX mice, which we derived directly from published evidence (Valko et al., 2013). Increasing the sample size of KO-ORX and WT mice above this estimation would have been questionable in light of the Reduction principle for animal experimentation (Tannenbaum and Bennett, 2015). On the other hand, we assumed the same effect size for the test of the hypothesis of fewer orexin neurons in KO-HIST than in WT mice, and our study could be underpowered if this assumption were violated. However, sample means of estimated total orexin neurons in KO-HIST and WT mice were very close, and raw neuron counts were, actually, higher in KO-HIST than in WT mice. Another limitation of our study is that it rests upon the assumption of normality in the distributions of neuron counts. This assumption was shared by previous work on the topic (John et al., 2013; Valko et al., 2013) and was not contradicted by our own statistical tests of normality, although this latter finding should be viewed with caution due to low sample size. The ORX-KO mice that we studied were slightly younger than the WT mice, although the age difference was not statistically significant due to variability, particularly in the WT group. However, age did not correlate significantly with HDC-positive neuron count in either group. We investigated differences in neuron number only with immunohistochemistry, following the example of previous work on this topic (John et al., 2013; Valko et al., 2013). A recognized limitation of anti-HDC antibodies for immunohistochemistry (Mizuguchi et al., 1990; Valko et al., 2013) is that they may also stain some monoaminergic neurons due to cross-reactivity. However, we took great care to count HDC-positive neurons within mouse TMN boundaries, which do not overlap with those of monoaminergic nuclei on the coronal plane (Franklin and Paxinos, 2007), and are not reported to include monoaminergic neurons (Haas et al., 2008). Future investigations should attempt to quantify not only the number of HDC- and orexin-positive neurons, but also the neuronal expression of HDC and orexin, employing techniques such as Western blotting (Krusong et al., 2011), retrotranscription with real-time polymerase chain reaction, and immunohistochemistry (Kalogiannis et al., 2010). Besides, we did not measure brain histamine levels. Recent, evaluations indicated that the KO-HIST mice lack histamine at least at the level of the neocortex, hypothalamus, and striatum (Castellan Baldan et al., 2014). However, the original report on the generation of KO-HIST mice reported that their brain histamine levels only fell by approximately 69% compared to those of WT controls (Ohtsu et al., 2001). The reasons for this discrepancy and the sources of any residual brain histamine in KO-HIST mice remain unclear, but residual histamine appears to be non-neuronal and outside the blood brain barrier because no immunoreactivity to either HDC or histamine was detected in the KO-HIST TMN and throughout their whole brain in this and previous studies (Parmentier et al., 2002).

Finally, our study is descriptive and confirms the previous published report on KO-ORX (Valko et al., 2013) with no additional insight into why there are more detectable histaminergic neurons in KO-ORX mice.

In conclusion, our data support the view that the loss of orexin peptides, as opposed to the loss of orexin cotransmitters or to the sequelae of the hypothalamic autoimmune insult hypothesized to trigger NT1, is sufficient to increase the number of histamine neurons in mice. Conversely, our data do not support the view that histamine is required for orexin neuron development in mice.

## Data Availability Statement

The raw data supporting the conclusions of this article will be made available by the authors, without undue reservation.

## Ethics Statement

The animal study was reviewed and approved by Italian Ministry of Health (protocols no. 245/2015-PR and 141/2018-PR).

## Author Contributions

CBe, AS, and GZ study design. SB, SA, GM, CBe, and VLM animal husbandry, surgery, and recordings. CBe, VLM, and CBo immunohistochemistry and neuron counting. CBe, AS, HO, J-SL, and GZ data analysis and interpretation. CBe and VLM manuscript draft. All authors reviewed the manuscript for important intellectual content.

## Conflict of Interest

The authors declare that the research was conducted in the absence of any commercial or financial relationships that could be construed as a potential conflict of interest.

## Footnotes

1 https://imagej.nih.gov/ij/

## References

[B1] Abercrombie M. (1946). Estimation of nuclear population from microtome sections. *Anat. Rec.* 94 239–247. 10.1002/ar.1090940210 21015608

[B2] Bastianini S. Silvani A. Berteotti C. Elghozi J. L. Franzini C. Lenzi P. (2011). Sleep related changes in blood pressure in hypocretin-deficient narcoleptic mice. *Sleep* 34 213–218. 10.1093/sleep/34.2.213 21286242 PMC3022942

[B3] Bastianini S. Silvani A. Berteotti C. Lo Martire V. Cohen G. Ohtsu H. (2015). Histamine transmission modulates the phenotype of murine narcolepsy caused by orexin neuron deficiency. *PLoS One* 10:e0140520. 10.1371/journal.pone.0140520 26474479 PMC4608736

[B4] Bonnavion P. Mickelsen L. E. Fujita A. de Lecea L. Jackson A. C. (2016). Hubs and spokes of the lateral hypothalamus: cell types, circuits and behaviour. *J. Physiol.* 594 6443–6462. 10.1113/JP271946 27302606 PMC5108896

[B5] Castellan Baldan L. Williams K. A. Gallezot J. D. Pogorelov V. Rapanelli M. Crowley M. (2014). Histidine decarboxylase deficiency causes tourette syndrome: parallel findings in humans and mice. *Neuron* 81 77–90. 10.1016/j.neuron.2013.10.052 24411733 PMC3894588

[B6] Chemelli R. M. Willie J. T. Sinton C. M. Elmquist J. K. Scammell T. Lee C. (1999). Narcolepsy in orexin knockout mice: molecular genetics of sleep regulation. *Cell* 98 437–451. 10.1016/s0092-8674(00)81973-x 10481909

[B7] Chen A. Singh C. Oikonomou G. Prober D. A. (2017). Genetic analysis of histamine signaling in larval zebrafish sleep. *eNeuro* 4:ENEURO.0286-16.2017. 10.1523/ENEURO.0286-16.2017 28275716 PMC5334454

[B8] de Lecea L. Kilduff T. S. Peyron C. Gao X.-B. Foye P. E. Danielson P. E. (1998). The hypocretins: hypothalamus-specific peptides with neuroexcitatory activity. *Proc. Natl. Acad. Sci. U.S.A.* 95 322–327. 10.1073/pnas.95.1.322 9419374 PMC18213

[B9] Eriksson K. S. Sergeeva O. Brown R. E. Haas H. L. (2001). Orexin/hypocretin excites the histaminergic neurons of the tuberomammillary nucleus. *J. Neurosci.* 21 9273–9279. 10.1523/JNEUROSCI.21-23-09273.2001 11717361 PMC6763926

[B10] Faul F. Erdfelder E. Lang A. G. Buchner A. (2007). G^∗^Power 3: a flexible statistical power analysis program for the social, behavioral, and biomedical sciences. *Behav. Res. Methods* 39 175–191. 10.3758/bf03193146 17695343

[B11] Franco P. Dauvilliers Y. Inocente C. O. Guyon A. Villanueva C. Raverot V. (2019). Impaired histaminergic neurotransmission in children with narcolepsy type 1. *CNS Neurosci. Ther.* 25 386–395. 10.1111/cns.13057 30225986 PMC6488909

[B12] Franklin B. J. K. Paxinos G. (2007). *The Mouse Brain in Stereotaxic Coordinates*, 3rd Edn. New York, NY: Academic Press.

[B13] Haas H. L. Sergeeva O. A. Selbach O. (2008). Histamine in the nervous system. *Physiol. Rev.* 88 1183–1241. 10.1152/physrev.00043.2007 18626069

[B14] Hyeun J. A. Kim J. Y. Kim C. H. Kim J. H. Lee E. Y. Seo J. H. (2019). Iron is responsible for production of reactive oxygen species regulating vasopressin expression in the mouse paraventricular nucleus. *Neurochem. Res.* 44 1201–1213. 10.1007/s11064-019-02764-x 30830595

[B15] John J. Thannickal T. C. McGregor R. Ramanathan L. Ohtsu H. Nishino S. (2013). Greatly increased numbers of histamine cells in human narcolepsy with cataplexy. *Ann. Neurol.* 74 786–793. 10.1002/ana.23968 23821583 PMC8211429

[B16] Kalogiannis M. Grupke S. L. Potter P. E. Edwards J. G. Chemelli R. M. Kisanuki Y. Y. (2010). Narcoleptic orexin receptor knockout mice express enhanced cholinergic properties in laterodorsal tegmental neurons. *Eur. J. Neurosci.* 32 130–142. 10.1111/j.1460-9568.2010.07259.x 20576035 PMC2945818

[B17] Kornum B. R. (2020). Narcolepsy Type 1: what have we learned from immunology? *Sleep* 43:zsaa055. 10.1093/sleep/zsaa055 32227223

[B18] Krusong K. Ercan-Sencicek R. Xu M. Ohtsu H. Anderson G. M. State M. W. (2011). High levels of histidine decarboxylase in the striatum of mice and rats. *Neurosci. Lett.* 495 110–114. 10.1016/j.neulet.2011.03.050 21440039 PMC3081964

[B19] Lemus M. B. Bayliss J. A. Lockie S. H. Santos V. V. Reichenbach A. Stark R. (2015). A stereological analysis of NPY, POMC, orexin, GFAP astrocyte, and Iba1 microglia cell number and volume in diet-induced obese male mice. *Endocrinology* 156 1701–1713. 10.1210/en.2014-1961 25742051

[B20] Lin L. Wisor J. Shiba T. Taheri S. Yanai K. Wurts S. (2002). Measurement of hypocretin/orexin content in the mouse brain using an enzyme immunoassay: the effect of circadian time, age and genetic background. *Peptides* 23 2203–2211. 10.1016/s0196-9781(02)00251-6 12535700

[B21] McGregor R. Shan L. Wu M. F. Siegel J. M. (2017). Diurnal fluctuation in the number of hypocretin/orexin and histamine producing: implication for understanding and treating neuronal loss. *PLoS One* 12:e0178573. 10.1371/journal.pone.0178573 28570646 PMC5453544

[B22] Mizuguchi H. Yabumoto M. Imamura I. Fukui H. Wada H. (1990). Immuno-cross-reactivity of histidine and dopa decarboxylases. *Biochem. Biophys. Res. Commun.* 173 1299–1303. 10.1016/s0006-291x(05)80928-3 2268331

[B23] Ohtsu H. Tanaka S. Terui T. Hori Y. Makabe-Kobayashi Y. Pejler G. (2001). Mice lacking histidine decarboxylase exhibit abnormal mast cells. *FEBS Lett.* 502 53–56. 10.1016/s0014-5793(01)02663-1 11478947

[B24] Parmentier R. Ohtsu H. Djebbara-Hannas Z. Valatx J. L. Watanabe T. Lin J. S. (2002). Anatomical, physiological, and pharmacological characteristics of histidine decarboxylase knock-out mice: evidence for the role of brain histamine in behavioral and sleep-wake control. *J. Neurosci.* 22 7695–7711. 10.1523/JNEUROSCI.22-17-07695.2002 12196593 PMC6757981

[B25] Peyron C. Faraco J. Rogers W. Ripley B. Overeem S. Charnay Y. (2000). A mutation in a case of early onset narcolepsy and a generalized absence of hypocretin peptides in human narcoleptic brains. *Nat. Med.* 6 991–997. 10.1038/79690 10973318

[B26] Sakurai T. Amemiya A. Ishii M. Matsuzaki I. Chemelli R. M. Tanaka H. (1998). Orexins and orexin receptors: a family of hypothalamic neuropeptides and G protein-coupled receptors that regulate feeding behavior. *Cell* 92 573–585. 10.1016/S0092-8674(00)80949-6 9491897

[B27] Sundvik M. Kudo H. Toivonen P. Rozov S. Chen Y. C. Panula P. (2011). The histaminergic system regulates wakefulness and orexin/hypocretin neuron development via histamine receptor H1 in zebrafish. *FASEB J.* 25 4338–4347. 10.1096/fj.11-188268 21885652

[B28] Sundvik M. Panula P. (2015). Interactions of the orexin/hypocretin neurones and the histaminergic system. *Acta Physiol. (Oxf)* 213 321–333. 10.1111/apha.12432 25484194

[B29] Tannenbaum J. Bennett B. T. (2015). Russell and Burch’s 3Rs then and now: the need for clarity in definition and purpose. *J. Am. Assoc. Lab. Anim. Sci.* 54 120–132. 25836957 PMC4382615

[B30] Thannickal T. C. Moore R. Y. Nienhuis R. Ramanathan L. Gulyani S. Aldrich M. (2000). Reduced number of hypocretin neurons in human narcolepsy. *Neuron* 27 469–474. 10.1016/s0896-6273(00)00058-1 11055430 PMC8760623

[B31] Valko P. O. Gavrilov Y. V. Yamamoto M. Reddy H. Haybaeck J. Mignot E. (2013). Increase of histaminergic tuberomammillary neurons in narcolepsy. *Ann. Neurol.* 74 794–804. 10.1002/ana.24019 24006291

